# Influence of Epoxidized Cardanol Functionality and Reactivity on Network Formation and Properties

**DOI:** 10.3390/polym12091956

**Published:** 2020-08-29

**Authors:** Emre Kinaci, Erde Can, John J. La Scala, Giuseppe R. Palmese

**Affiliations:** 1 Department of Chemical and Biological Engineering, Drexel University, Philadelphia, PA 19104, USA; peveenrete@gmail.com; 2 Department of Chemical Engineering, Faculty of Engineering, Yeditepe University, 34755 Ataşehir, Istanbul, Turkey; erde.can@yeditepe.edu.tr; 3 Army Research Laboratory, 4600 Deer Creek Loop, Aberdeen Proving Grounds, MD 21005-5096, USA; john.j.lascala.civ@mail.mil

**Keywords:** biobased, epoxy, thermoset, cardanol, reactivity, cure

## Abstract

Cardanol is a renewable resource based on cashew nut shell liquid (CNSL), which consists of a phenol ring with a C15 long aliphatic side chain in the meta position with varying degrees of unsaturation. Cardanol glycidyl ether was chemically modified to form side-chain epoxidized cardanol glycidyl ether (SCECGE) with an average epoxy functionality of 2.45 per molecule and was cured with petroleum-based epoxy hardeners, 4-4′-methylenebis(cyclohexanamine) and diethylenetriamine, and a cardanol-based amine hardener. For comparison, cardanol-based diphenol diepoxy resin, NC514 (Cardolite), and a petroleum-based epoxy resin, diglycidyl ether of bisphenol-A (DGEBA) were also evaluated. Chemical and thermomechanical analyses showed that for SCECGE resins, incomplete cure of the secondary epoxides led to reduced cross-link density, reduced thermal stability, and reduced elongation at break when compared with difunctional resins containing only primary epoxides. However, because of functionality greater than two, amine-cured SCECGE produced a *T*_g_ very similar to that of NC514 and thus could be useful in formulating epoxy with renewable cardanol content.

## 1. Introduction

Thermosetting polymers are being used extensively in coatings, adhesives, and as matrices in polymer composites because of their good mechanical properties, remarkable chemical and thermal resistance, as well as good processability [1,2]. Renewable thermosets derived from biomass and designed using the unique chemical and structural characteristics of natural building blocks could offer many sustainable alternatives to incumbent systems based on improved performance. An important class of biobased molecules that can be functionalized and used in the synthesis of thermosets is derived from cashew nutshell liquid (CNSL). Cardanol, which is the main component of the thermally treated CNSL, is a phenolic lipid with C15 alkyl chains at the meta position. Cardanol contains a mixture of saturated (~5%), mono- (~40%), di- (~20%) and, tri- (~35%) unsaturated alkyl side chains with unsaturation sites at the 8–9th, 11–12th, and 14–15th carbon positions of the chain [3,4]. The unique structure of cardanol, which combines a rigid phenol ring with a flexible C15 alkyl chain, makes it a promising candidate for applications that require a combination of rigidity, chemical resistance, and water barrier properties [5].

Modification of cardanol for thermoset synthesis has been achieved by the functionalization of the phenolic ring as well as the alkyl side chain. In previous work, cardanol has been functionalized at the phenol moiety to synthesize monofunctional epoxy using epichlorohydrin. This molecule can be used as a chain-capping agent in thermoset formulations [6,7,8]. Functionalization of the cardanol at the unsaturation sites of the side chain has also been performed via phenolation of the double bonds [9]. Once the cardanol molecule is phenolated, subsequent epoxidation using epichlorohydrin produces monomers useful for thermosetting materials [7,10,11,12]. In addition to phenolation, chemical modification of the side chain via direct epoxidation of the double bonds is possible. One of the most common, low-cost, and sustainable methods to epoxidize the olefinic compounds is to utilize peroxy acids created in situ from the reaction of formic acid and hydrogen peroxide [13]. Other methods such as enzymatic epoxidation [14] and epoxidation with strong acids such as meta-chloroperbenzoic acid [15] (m-CPBA) have also been used to functionalize the side chain of the cardanol to create secondary, aliphatic epoxies.

Previous studies of the reactivity of epoxidized soy-bean oil (ESO) and its derivatives showed that secondary epoxies are not as reactive as primary glycidyl moieties and cannot be effectively cured to a high extent with amine curing agents [16,17,18]. In such systems, incomplete cure at stoichiometry results in the reduced cross-link density of the network and the loss of important network properties such as mechanical and thermomechanical resistance [19,20]. Thus, it is important to be able to quantify the extent of curing of secondary epoxies in order to obtain well-defined structure−property relations.

Herein, functionalization of the unsaturation sites of the cardanol glycidyl ether (CGE) side chain was achieved via hydrogen peroxide and formic acid to obtain side chain epoxidized CGE (SCECGE) with secondary epoxies on the side chain. Structural verification of the SCECGE monomer was obtained using proton nuclear magnetic resonance (^1^H NMR) and epoxy equivalent weight (EEW) titrations. Earlier studies concerning the epoxidation of primary unsaturation sites show that formic acid/hydrogen peroxide methods are not effective for epoxidizing terminal double bonds [21,22,23,24]. In this study, a detailed structural analysis was performed to determine the extent of internal and terminal double bond epoxidation of SCECGE monomer. Furthermore, the extent of curing reactions for different SCECGE epoxy-amine combinations were also investigated using a number of direct techniques to monitor the extent of cure and reactivity of the secondary epoxy moieties versus that of the glycidyl epoxies by using a cardanol-derived secondary epoxy compound model. Thermomechanical and mechanical properties of the postcured networks were evaluated to assess structure−property relationships of this material and to link these properties to the degree of epoxy cure.

## 2. Materials and Methods

### 2.1. Materials

**Figure 1:**
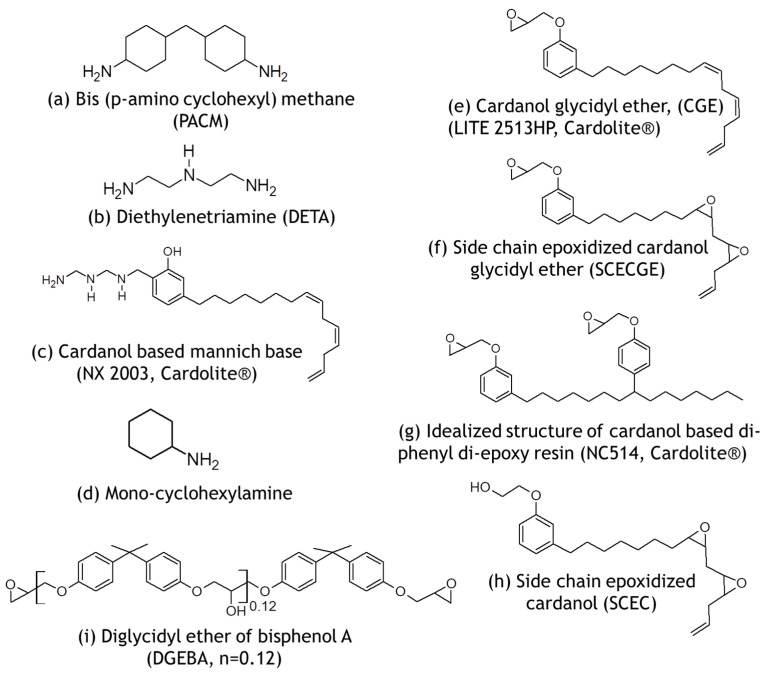
Representative structures of the materials used in this study.

Formic acid (FA, 99%), toluene (99.8%), hydrogen peroxide (H_2_O_2_, 30 wt.%), diethylenetriamine (DETA, 99.9%), sodium chloride (NaCl, 99.9%), sodium bicarbonate (99.9%), magnesium sulfate (99.9%), and monocyclohexylamine (MHA, 99.9%, AHEW: 55 g/equivalent) were obtained from Sigma-Aldrich, USA; 4-4′-methylenebis(cyclohexanamine) (PACM, AHEW: 52.5 g/equivalent) was obtained from Air Products, Allentown, PA, USA. Cardanol-based di-phenyl di-epoxy (NC514), mono-epoxy, cardanol glycidyl ether (CGE, LITE2513 HP) and cardanol-based epoxy hardener NX2003 (AHEW: 95 g/equivalent) were kindly supplied by Cardolite, Newark, NJ, USA. All chemicals in this work were used as-received. Structures of the primary materials used in this work are presented in Figure 1. It is important to note that the amine hydrogen equivalent weight (AHEW) values of the epoxy hardeners were obtained from the material specification sheet.

### 2.2. Preparation of SCECGE Resin

**Scheme 1:**
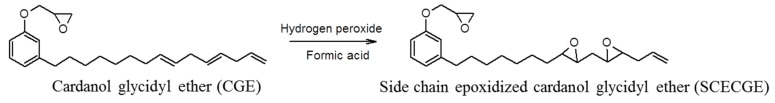
Representative reaction scheme for the synthesis of SCECGE.

The double bonds of the monoepoxy CGE were epoxidized using peroxy acids, which are produced from FA and H_2_O_2_ in situ. To maintain safe reaction conditions and prevent thermal runaway, the epoxidation reactions were carried out at a low temperature and low peroxy acid concentrations. Scheme 1 shows the representative reaction scheme for the epoxidation of CGE to produce the SCECGE resin. In a representative procedure, CGE monomer (65 g, 1 mol of double bonds) was charged into a 2-L round-bottom flask together with FA (46.1 g, 1 mol) and toluene (260 g, 2.5 mol) under constant stirring. H_2_O_2_ (30 wt.%) (340 g, 10 mol) was added to the flask drop-wise over 1 h at 20 °C. After the addition was completed, the temperature of the system was slowly increased to 40 °C over 1 h, and the contents of the reaction mixture were allowed to react at that temperature for 24 h. At the end of the reaction time, the contents were washed with 5 wt.% sodium bicarbonate solution followed by deionized water to remove the excess acid and 5 wt.% NaCl solution three times. The remaining residue was dried with anhydrous magnesium sulfate and rotary evaporated to yield a brownish-yellow liquid product (90% yield).

### 2.3. Characterization of the SCECGE Monomer

EEW titrations were performed in accordance with the ASTDM D1652-97 standard to determine the epoxy content of the CGE, SCECGE, as well as DGEBA and NC514 epoxies. ^1^H NMR spectroscopy was used for characterization of reactants and products. A ^1^H-NMR (500 MHz, Varian Unity Inova, Palo Alto, CA, USA) unit was used with a spectral window of ±2000 Hz, 90° pulse width, and 32 scans at 25 °C. The epoxide functionality of NC514 and DGEBA were obtained using the ^1^H NMR method of Jaillet et al. [10] These were found to be 1.35 per phenol group on NC514, which corresponds to 2.2 epoxies per average NC514 molecule and 2.00 per molecule of DGEBA. A TA Instruments AR2000 ex rheometer was used to determine the viscosity of the epoxy resins and epoxy hardeners at 25 °C with shear rates in the range of 2–200 s^−1^ using parallel plate geometry.

### 2.4. Preparation of Cured Samples of SCECGE, NC514, and DGEBA with Amine Curing Agents

Polymer samples were prepared by thermally cross-linking SCECGE, NC514, and DGEBA epoxy monomers with different petroleum-based (PACM and DETA) and cardanol-based (NX2003) epoxy hardeners at stoichiometry. The epoxy-amine combinations were mixed and degassed via a high shear mixer (Thinky ARE-310), cast into rectangular and dog-bone shaped rubber molds, and then thermally cured at 90 °C for 12 h and at 180 °C for 12 h. The conversion of epoxy and amine groups were measured using transmission near-IR in the 4000–8000 cm^−1^ range with an 8 cm^−1^ resolution and 32 scans per spectra at ambient conditions.

### 2.5. Model Compound Studies to Evaluate the Secondary Epoxy Reactivity

To determine the conversion of the secondary epoxies, cardanol-derived secondary epoxy containing model compound (SCEC) was utilized. The side chain epoxidation of the cardanol model compound was carried out using the same chemistry applied to functionalize the side chain of CGE molecule. The representative structure of this model compound is shown in Figure 1g. This monomer was cured with mono-cyclohexyl amine (MHA) at 100% molar excess amine. The purpose of using this epoxy-amine system was to prevent network formation so that the reaction products are soluble in solvents (i.e., deuterated chloroform and dichloromethane) used to obtain ^1^H NMR spectra and EEW of the mixture throughout the curing process. In addition, using SCEC instead of SCECGE monomer yielded clearer FT-IR and ^1^H-NMR spectra since the secondary epoxy peak of SCECGE overlaps with the primary epoxy peak, making sample analysis difficult. For mid-IR studies, the samples were sandwiched between inert NaCl and scanned with a resolution 8 cm^−1^ and 32 scans per spectrum. ^1^H-NMR and EEW studies were also used to determine the overall secondary epoxy conversion before and after the curing and postcuring steps.

In Equation (1), *ABS*(*t*) and *ABS*(*t* = 0) represent the reduced absorbance for the FTIR studies before and after the curing reaction, respectively. In Equation (2), *I*(*t*) and *I*(*t* = 0) also represent the reduced area of the ^1^H NMR peak after a certain time t, and the reduced peak area at the start of curing process. For the mid-IR studies, the secondary epoxy peak observed around 840 cm^−1^ was followed and referenced to the invariant aromatic peak at 1610 cm^−1^. For the ^1^H-NMR studies, the secondary epoxy peaks observed between 2.8–3.1 ppm were tracked and referenced to the invariant terminal methyl proton peak found at 0.88 ppm.

(1) $$\alpha_{2^{o}epoxy}=1-\left(\frac{ABS\left(t\right)840cm^{-1}}{ABS\;\left(t=0\right)840cm^{-1}}\right)\left(\frac{ABS\left(t=0\right)\;1610cm^{-1}}{ABS\left(t\right)\;1610cm^{-1}}\right)$$

(2) $$\alpha_{2^{o}epoxy}=1-\left(\frac{I\left(t\right)2.8-3.1ppm}{I\left(t=0\right)2.8-3.1ppm}\right)\left(\frac{I\left(t=0\right)\;0.88ppm}{I\left(t\right)\;0.88ppm}\right)$$

### 2.6. Properties of the Cured Network

#### 2.6.1. Thermomechanical Analysis via Dynamic Mechanical Analysis (DMA)

Dynamic mechanical analysis (DMA, TA Instruments, Q800) was used to evaluate the cross-link density (*v*), glass transition temperature (*T*_g_), as well as the room-temperature storage modulus (*E*′) of the cured polymers. Rectangular samples with approximate dimensions of 35.5 × 12 × 1.0 mm^3^ were tested isothermally at 25 °C and 1 Hz with a deflection of 15 µm by using a single cantilever arrangement to obtain *E*′ accurately. To determine the *T*_g_ and calculate cross-link density of the cured samples, rectangular DMA bars with approximate dimensions of 35.5 × 12.0 × 3.5 mm^3^ were tested from −100 °C to well above their glass transition temperature by ramping at 2 °C/min at 1 Hz with a deflection of 15 µm. The temperature value of the maximum of the loss modulus peak was taken as the *T*_g_ of the cured polymer. The *v* values were calculated from the rubbery plateau of the storage modulus curves at 50 °C above *T*_g_ using Equation (3) where R is the universal gas constant [25].

(3) $$v=E^{\prime }/3RT$$

#### 2.6.2. Mechanical Properties via Tensile Tests

The mechanical properties of the cured samples were determined via tensile tests in accordance with the ASTM D-638 standard test method using an Instron UTM (8800). Tensile tests were performed on type IV samples. Samples were tested at ambient conditions using an extensometer to measure strain with a constant crosshead speed of 1 mm/min and a gauge length of 45 mm. For each formulation, at least 6 tensile specimens were tested and analyzed.

## 3. Results and Discussion

### 3.1. Monomer Characterization

**Figure 2:**
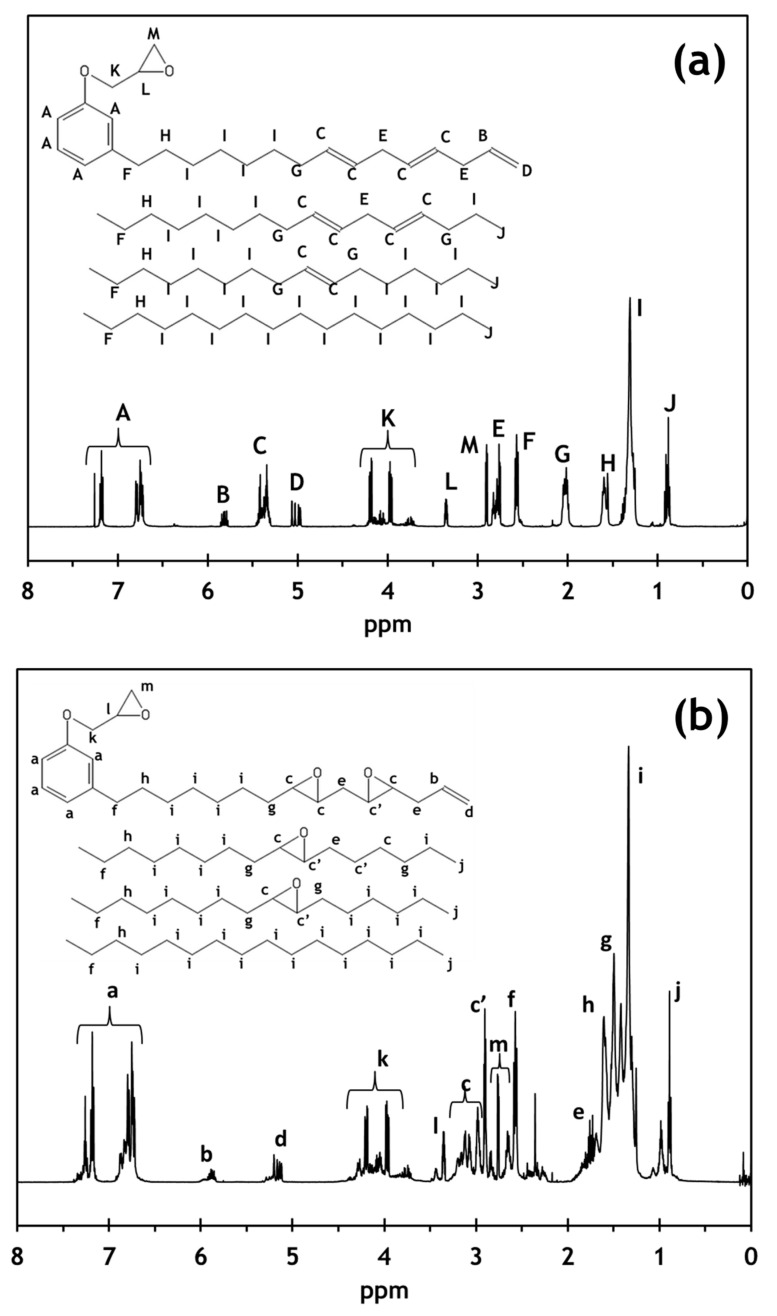
^1^H-NMR analysis of the reactant (**a**-top) and the epoxidized product (**b**-bottom). Chemical shift assignments are provided in Table S1 of the supplementary information.

Figure 2a,b show the ^1^H-NMR spectra of CGE and its epoxidation product SCECGE with the corresponding peak assignments for the different types of protons of CGE and SCECGE, respectively. The epoxidation of the side chain was confirmed by the shifting of the peak at 5.3 ppm, corresponding to the methylene protons (–CH=CH–) in the spectrum of CGE to 3.2–3.0 and 2.95 ppm (c and c′), representing the secondary epoxy protons on the side chain. In addition, the peak at 2.8 8 ppm (E) assigned to the protons between two double bonds (–CH=CH–CH_2_–CH=CH–) in the spectrum of CGE shifted to 1.73–1.91 91 ppm (e peak), representing the protons between the two epoxies, and G peak at 2.00 ppm (–CH_2_ protons next to double bonds) present in the spectrum of CGE shifted to 1.5 ppm (g) in the spectrum of SCECGE, which was attributed to the protons next to the epoxy ring. More detailed ^1^H NMR and ^12^C NMR analysis of the CGE and SCECGE polyepoxy monomer can be found elsewhere [14,15].

The double bond and epoxy functionalities of the CGE were determined via the integration of the related ^1^H NMR peaks. The four aromatic protons of (A) were used as the internal invariant. The internal and terminal double bond functionality of the CGE was determined as 1.45 and 0.38 double bond/molecule, respectively, as shown in Table 1, while the primary epoxy functionality of the CGE was calculated as 0.99 epoxy/molecule. After the 24 h of reaction, the internal double bond functionality of the SCECGE reduced to zero, meaning that nearly all the internal double bonds were converted to the secondary epoxies (a small percent form open epoxies or are lost through other side reactions); thus, the secondary epoxy functionality of the SCECGE molecule was calculated as 1.40 epoxy/molecule. The terminal double bonds of the CGE molecule were not effectively epoxidized. A slight increase from 0.99 to 1.05 primary epoxy/molecule was observed after 24 h of reaction, and the terminal double bond functionality was reduced from the 0.38 to 0.32. Thus, SCECGE resin has a total of 2.45 epoxy/molecule, where 1.05 is primary (phenolic and terminal epoxy) and 1.40 is aliphatic secondary epoxies. Similarly, EEW values of CGE resin decreased from 387 g/equivalent to 177 g/equivalent for SCECGE, supporting the addition of the oxirane rings on the side chain was successful, as summarized in Table 1. An investigation of the terminal double bond reactivity and its epoxidation will be the subject of a future communication.

Detailed structure analysis previously performed on cardanol-based NC514 epoxy resin showed that this resin contains a significant amount of impurities in the form of higher molecular weight oligomers formed during the phenolation and epoxidation of cardanol [10,12]. Structural analysis of NC514 monomer was also performed to calculate the epoxy functionality by using the ^1^H NMR spectra of the cardanol-based di-phenyl di-epoxy NC514. The epoxy functionality of the NC514 epoxy resin was calculated as 1.35 epoxy/cardanol unit via its ^1^H NMR spectra. A minor amount of unreacted cardanol (~4%) was also observed in NC514. In addition, size exclusion chromatography analysis of this resin showed that NC514 is effectively a mixture of phenolated, epoxidized cardanol and higher molecular weight species, as determined previously [7]. The higher molecular weight species is due to the di-substitution on the phenol ring that occurs during the phenolation of cardanol, leading to di-, tri-, and multimere structures with an increase of the molar mass, as explained by Ionescu et al. [9]. The EEW of the cardanol-based NC514 epoxy was determined as 440 g/equivalent (Table 1), which is significantly higher than the theoretical value of ~375 g/equivalent that is determined via the structure proposed by the supplier; this is due to the higher molecular weight oligomers present in the mixture that increase the EEW. In fact, the molecular weight of the NC514 resin can be as high as ~1000 g/mol as determined via gel permeation chromatography [10,12], suggesting that NC514 has around 2.2 epoxies per oligomer but with only 1.35 epoxies per cardanol unit.

Viscosity measurements were a performed on NC514, DGEBA, CGE, and SCECGE resins at 25 °C. The viscosity of all the epoxy resins did not show significant dependence of shear rate, suggesting Newtonian behavior. Viscosity values—measured at 20 s^−1^ shear rate, given in Table 1—show that the side-chain epoxidation reaction more than doubled the viscosity of the CGE resin from 0.15 to 0.33 Pa s. This increase in viscosity was attributed to the addition of the epoxy moiety on the side chain, which reduces side-chain mobility and increases polarity.

### 3.2. Curing of the Epoxy Monomers with Different Amine Curing Agents

**Figure 3:**
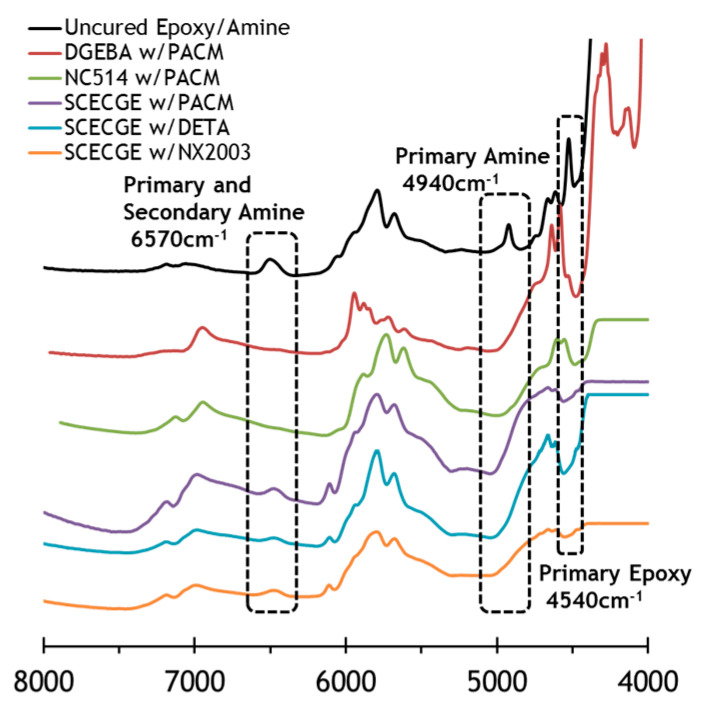
Near-IR spectra of the different epoxy-amine formulations after postcure. For comparison, the uncured epoxy-amine spectrum is included.

Figure 3 shows the near-IR spectra of representative cured resin formulations versus that of an uncured resin. No traces of the primary amine peak at 4940 cm^−1^, the primary and secondary amine peak at 6570 cm^−1^, or the epoxy peak at 4540 cm^−1^ were observed in the cured samples of DGEBA and NC514. The absence of these peaks indicates a complete epoxy/amine reaction in the cured samples within the limits of IR spectroscopy measurements. For SCECGE, no primary amine peak was observed after cure either. However, even after the prolonged curing times at elevated temperatures, a slight peak at 6570 cm^−1^ associated with primary and secondary amine was still observed, suggesting that SCECGE-based samples were not cured fully as was the case for DGEBA and NC514. The incomplete curing of SCECGE-based samples may be explained by the lower reactive nature of the secondary epoxies presented on the molecule, which do not react easily with the secondary amines to form a highly cross-linked network, as demonstrated by previous studies. Although this extent of conversion would indicate that the conversion of the secondary epoxies is around 60–70% for different curing agents—assuming complete conversion of the glycidyl primary epoxides—there are no distinct peaks visible for the secondary epoxies in near-IR, so this method does not allow for direct measurement. Thus, the investigation with side chain epoxidized model compound (SCEC) described in the next section was designed to evaluate the conversion of the secondary epoxies with amines at temperatures typical of the cure and postcure of epoxy-amines.

### 3.3. Extent of Secondary Epoxy Cure via Model Compound Studies

Although near-IR can provide valuable information about the curing reaction between epoxies and amine systems, it is not possible to specifically monitor the secondary epoxies with this technique. To determine the conversion of the secondary epoxies and the extent of the cure reaction between secondary epoxy and amines, SCEC (EEW: 275 g/eq) model epoxy was cured with the monoamine MHA in 100% molar excess. The secondary epoxy and amine reaction was followed via near-IR, ^1^H-NMR, and EEW titrations as described in the experimental section.

**Figure 4:**
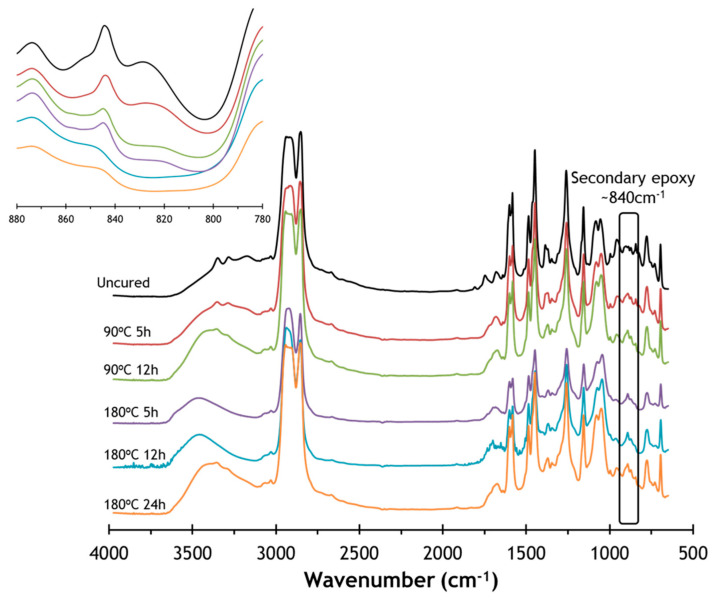
Mid-IR spectra of the SCEC-MHA mixture with 100% excess amine with respect to curing time together with magnified spectra of the secondary epoxies around 840 cm^−1^.

**Figure 5:**
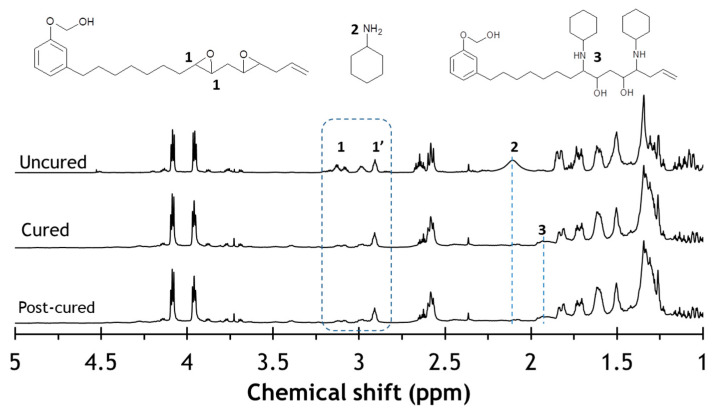
^1^H NMR spectra of the SCEC-MHA epoxy-amine mixture with 100% excess amine: initial uncured spectrum; spectrum of material cured at 90 °C for 12 h; and spectrum of material postcured at 180 °C for 24 h.

Figure 4 shows the mid-IR spectra of the SCEC-MHA system after curing and postcuring steps along with the initial uncured spectrum. The peak observed around 840 cm^−1^ shows that the secondary epoxy reduced in intensity during the curing process. However, even after prolonged curing times at elevated temperatures, this peak did not completely disappear, suggesting the secondary epoxies were not fully converted. The conversion of the secondary epoxies was determined to be 60% after 12 h of curing at 90 °C and increased to 72% after postcuring at 180 °C for 24 h. Postcuring the mixture for longer times did not increase the conversion any further. In addition, representative ^1^H-NMR spectra of the SCEC-MHA mixture are presented in Figure 5 after the curing and postcuring steps along with the initial uncured spectrum. The intensity of the secondary epoxy peaks designated as (1) and (1′), observed between 2.9–3.2 ppm, reduced in intensity after the curing and postcuring steps but did not change any further after 24 h. In addition, the intensity of the primary amine peak found around 2.7 ppm (2) also reduced greatly but did not fully disappear. A secondary amine peak was formed around 1.70 ppm (3) during cure and remained after postcure. The conversion of the secondary epoxies was determined as 45% after curing and 65% after the specified postcuring cycle at 180 °C. The EEW of the SCEC-MHA mixture after the curing and postcuring steps was determined via titrations. The initial EEW of the SCEC-MHA mixture was found to be 360 g/eq. Following cure at 90 °C, the EEW increased to 710 g/eq, and after the postcuring, the EEW further increased to 970 g/eq, suggesting that 63% of the secondary epoxies reacted after the postcure. In summary, three distinct methodologies were applied to monitor the conversion of the secondary epoxies with a monoamine and showed a secondary epoxy conversion between 63–72%. These results are consistent with the calculated secondary epoxy conversion for the SCECGE-based samples described in the previous section and suggest a limit to the overall conversion of the secondary epoxies in question.

### 3.4. Extent of Secondary Epoxy Cure via Model Compound Studies

#### 3.4.1. Dynamic Mechanical Analysis of the Cured Samples

**Figure 6:**
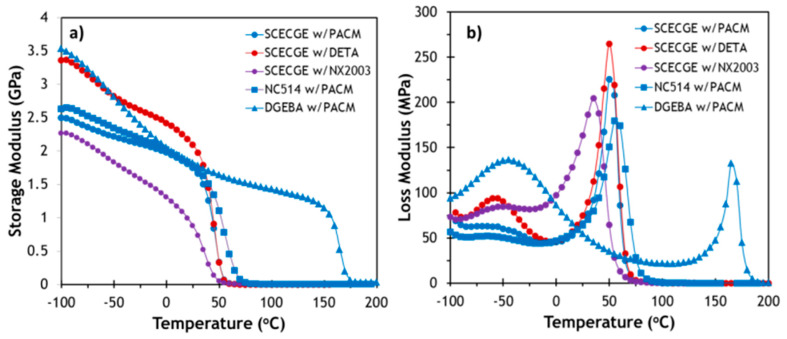
(**a**) Storage and (**b**) loss modulus variations with temperature for the cured samples of SCECGE resins cured with the listed epoxy hardeners. For comparison, DGEBA and NC514 epoxy resins cured with PACM are included.

Figure 6a,b show the temperature dependence of the storage modulus and loss modulus for SCECGE epoxy resin cured with the listed amine curing agents. For comparison, NC514 and DGEBA resins cured with PACM are also presented in Figure 6. Table 2 lists the values of the cross-link density of the epoxy/amine formulations obtained as described in the experimental section. The cross-link density of DGEBA-based networks showed the highest value among the epoxy resins for all of the amine curing agents used. DGEBA has a lower EEW than that of NC514, so this result was expected. On the other hand, Table 2 also shows that the NC514-based formulations have higher cross-link density than the SCECGE-based networks despite the higher epoxy functionality and lower EEW of SCECGE. This is explained by the fact that SCECGE-based formulations cannot be cured to as high an extent as NC514-based formulations because of the less reactive nature of the secondary epoxies of the SCECGE molecule. The cross-link density of the SCECGE-based formulations using various curing agents was also estimated via the method proposed by Hill et al. using 60% secondary epoxy conversion [26]. The estimated cross-link density values had good agreement with the values calculated via DMA measurements, which were indeed much lower than the theoretical 100% conversion cross-link density values. A detailed explanation of the cross-link density calculation via Hill’s method is presented in the supplemental information section (Tables S2 and S3). Polymers prepared with DETA had higher cross-link densities than PACM due to the lower AHEW values for DETA. The cardanol-based NX2003 has the same number of reactive amino sites as PACM, but has a higher molecular weight, which results in the lowest cross-link density for the NX2003 networks, again following the trends as expected based on AHEW.

The glass transition temperature of the fully cured epoxy amine combinations presented in Table 2 show that the DGEBA-based formulations have a higher *T*_g_ compared to SCECGE and NC514 because of their higher cross-link density and rigid, aromatic backbone [27]. The values given in Table 2 also show the *T*_g_ of the NC514- and SCECGE-based formulations had significantly lower glass transition temperatures than the DGEBA-based systems, most likely because of the presence of the more flexible alkyl chain found between cross-links [27]. Furthermore, the reduction in the cross-link density for SCECGE—a reduction from what was expected based on a 100% extent of reaction—likely further reduces the *T*_g_ of this monomeric system. Formulations prepared with NC514 had only slightly higher glass transition temperatures than formulations prepared with SCECGE. A 5 °C difference in *T*_g_ was observed between the NC514 and SCECGE resins when cured with PACM. The *T*_g_ difference was 3 °C between the two monomers when cured with DETA. This is a surprisingly small difference considering the added higher aromatic content between cross-links found in networks formed with NC514 and the incomplete cure of the secondary epoxide groups in SCECGE. The reason for this could be related to an overall higher molecular weight between cross-links formed with NC514 and the nonreactive aliphatic side chain that is found these systems. Thus, the topological differences of the SCECGE networks relative to NC514 appear to narrow the presumed *T*_g_ gap associated with the greater aromatic content in NC514. Future studies aimed at epoxidizing the terminal double bonds of SCECGE will help to test this assertion.

Room-temperature storage modulus values were also determined via DMA by testing the rectangular DMA bars isothermally at 25 °C, and these results are also given in Table 2. The highest E’ values were observed for the DGEBA-based epoxy/amine systems because the cardanol-based resins were softening due to the relative closeness to their *T*_g_ (~25 °C from the *T*_g_, while the DGEBA samples were at least 70 °C below *T*_g_). In addition, these room temperature storage modulus values obtained via DMA are in a good agreement with the Young’s modulus values obtained via tensile tests, also shown in Table 2.

#### 3.4.2. Tensile Tests

**Figure 7:**
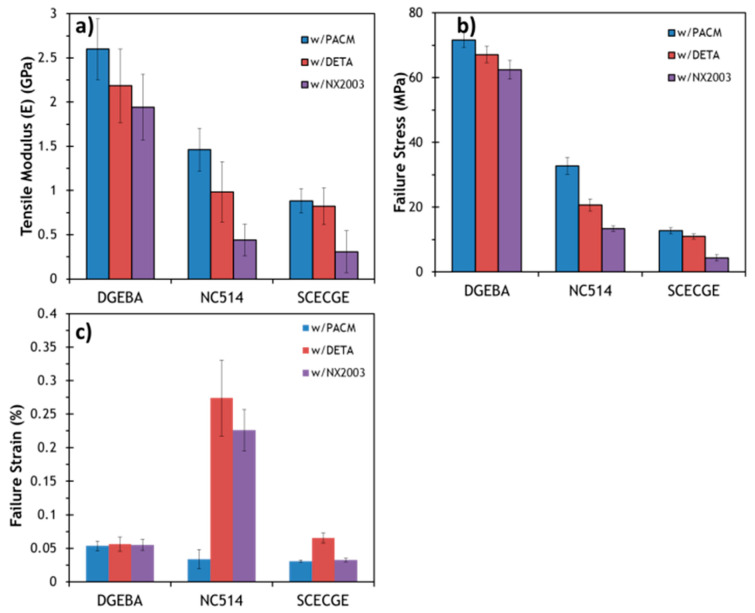
(**a**) Tensile modulus, (**b**) failure stress, and (**c**) failure strain of various cured samples obtained via tensile tests.

The tensile modulus (*E*), tensile strength, and tensile strain of the fully cured samples were obtained from tensile tests, and the results are shown in Figure 7 and Table 2. The highest tensile modulus and strength values, Figure 7a,b, were observed for the DGEBA samples because of the high cross-link density and rigid aromatic backbone of the polymer. The SCECGE and NC514 samples had lower tensile modulus and strength values because of the low rigidity of the C15 alkyl chain and lower cross-link densities of the polymer networks. The NC514 samples have slightly higher tensile modulus and strength relative to SCECGE due to the existence of the second phenyl ring on the polymer backbone of NC514.

The type of amine curing agent used has a significant effect on the modulus of the cured samples. PACM gave the highest tensile modulus and strength values despite their lower cross-link densities relative to DETA because of the stiffer nature of the cycloaliphatic amine PACM. Epoxy resins cured with the cardanol-based amine hardener NX2003 have shown the lowest tensile modulus and tensile strength values among the curing agents due to the plasticization effect of the C15 alkyl chain of the hardener and the lower cross-link densities for the NX2003 networks. This effect is more pronounced when the cardanol-based epoxies were cured with cardanol-based curing agents because of the large plasticization effect caused by the combination of the C15 alkyl chains, low extent of cure, and low cross-link density of the network. The elongation at break of the epoxy amine formulations presented in Figure 7c show that epoxy-amine formulations prepared with the cardanol-based NC514 had the highest values for elongation at break. Interestingly, the SCECGE-based formulations had significantly lower elongation at break values than the NC514-based formulations despite having similar aliphatic chain structure. These values were closer to the DGEBA-based formulation despite the flexible nature of SCECGE. This is explained by the incomplete secondary epoxy cure of the SCECGE-based formulations. As a result, there is a significant percentage of SCECGE cardanol units with only one cured epoxide, resulting in a chain-end defect that weakens the network. On the contrary, NC514 has high elongation since its epoxides react nearly completely and allow good load transfer across the cured resin.

## 4. Conclusions

The cardanol-based epoxy monomer SCECGE was prepared, and contained epoxy groups on the aliphatic side chain as well as the glycidyl ether on the phenolic ring. The structural characterization of SCECGE showed that epoxidation of the inner secondary double bonds was achieved almost completely, while the terminal ones were minimally epoxidized. Mid-IR analysis of the different epoxy-amine systems showed that the primary phenolic glycidyl ethers were almost fully reacting with the amine curing agents, but the secondary epoxies found on the SCECGE-based samples required a higher temperature for curing and were not observed to fully cure with the amines investigated. Model compound studies were used to evaluate secondary epoxy conversion by a number of methods, and it was found that for the reaction conditions investigated, it was possible to react between 60–70% of the secondary epoxy groups. Though it is well known that the secondary epoxide groups on aliphatic chains are significantly less reactive than glycidyl ethers, in this investigation the reactivity of these groups has been quantified, adding to our understanding of the cure of resin systems that incorporate these reactive groups. Results of the investigation showed that incomplete cure of the secondary epoxides led to a reduced cross-link density for SCECGE resins and reduced elongation at break. Otherwise, amine-cured SCECGE resin produced a *T*_g_ very similar to the cardanol-derived NC514 diepoxy, which contains only phenolic glycidyl ether reactive groups. Moreover, the viscosity of SCECGE is significantly lower than that of the other systems studied. Thus, SCECGE is a promising candidate for making useful epoxy resins with renewable content based on CNSL.

## Figures and Tables

**Table 1:** Properties of the epoxy monomers used in the study (* per oligomer).

Resin	Double Bond Functionality (per molecule)	Primary Epoxy Functionality (per molecule)	Secondary Epoxy Functionality (per molecule)	Total Epoxy Functionality (per molecule)	EEW (g/equivalent)	Viscosity (Pa s)
DGEBA	N/A	2.0	N/A	2.0	188	19.9
NC514	N/A	2.2 *	N/A	2.2 *	440	15.5
CGE	1.45 (internal) 0.38 (terminal)	0.99	N/A	0.99	378	0.15
SCECGE	0 (internal) 0.32 (terminal)	1.05	1.40	2.45	177	0.33

**Table 2:** Summary of the cured network properties (* per oligomer).

Sample	Crosslink Density (*v*) (mol/m^3^)	*T*g (°C) (DMA)	*E*′ (at 25 °C) (GPa)	Tensile Modulus (*E*) (GPa)	Failure Stress (MPa)	Failure Strain (mm/mm)
SCECGE w/PACM	375 ± 70	51	0.85	0.88 ± 0.1	12.7 ± 0.9	0.031 ± 0.002
SCECGE w/DETA	508 ± 61	50	0.89	0.82 ± 0.2	10.9 ± 0.8	0.065 ± 0.007
SCECGE w/NX2003	355 ± 40	36	0.34	0.32 ± 0.2	3.96 ± 0.4	0.061 ± 0.002
NC514 w/PACM	624 ± 67	56 *	1.48	1.46 ± 0.2	32.7 ± 2.6	0.033 ± 0.014
NC514 w/DETA	745 ± 130	53 *	0.88	0.98 ± 0.3	20.6 ± 1.9	0.274 ± 0.056
NC514 w/NX2003	388 ± 13	36 *	0.58	0.45 ± 0.2	10.4 ± 1.0	0.226 ± 0.030
DGEBA w/PACM	2553 ± 251	175	2.28	2.60 ± 0.3	71.6 ± 2.3	0.053 ± 0.007
DGEBA w/DETA	3630 ± 238	154	2.30	2.18 ± 0.4	67.1 ± 2.6	0.056 ± 0.01
DGEBA w/NX2003	1793 ± 140	94	1.86	1.94 ± 0.2	54.7 ± 1.6	0.054 ± 0.008

## Supplementary Materials

The following are available online at https://www.mdpi.com/2073-4360/12/9/1956/s1, Table S1: ^1^H-NMR chemical shift assignments for CGE and SCEGE, Table S2: Cross-link density values of SCECGE epoxy cured with different amines calculated via Hill’s method and DMA studies, and Table S3: Cross-link density values of DGEBA epoxy cured with different amines calculated via Hill’s method and DMA studies.

## References

[1] Dodiuk H., Goodman S.H. (2013). Handbook of Thermoset Plastics.

[2] Ratna D. (2009). Handbook of Thermoset Resins.

[3] Voirin C., Caillol S., Sadavarte N.V., Tawade B.V., Boutevin B., Wadgaonkar P.P. (2014). Functionalization of cardanol: Towards biobased polymers and additives. Polym. Chem..

[4] Tyman J. (1979). Non-isoprenoid long chain phenols. Chem. Soc. Rev..

[5] Verge P., Toniazzo V., Ruch D., Bomfim J.A.S. (2014). Unconventional plasticization threshold for a biobased bisphenol-a epoxy substitution candidate displaying improved adhesion and water-resistance. Ind. Crops Prod..

[6] Kanehashi S., Yokoyama K., Masuda R., Kidesaki T., Nagai K., Miyakoshi T. (2013). Preparation and characterization of cardanol-based epoxy resin for coating at room temperature curing. J. Appl. Polym. Sci..

[7] Can E., Kınacı E., Palmese G.R. (2015). Preparation and characterization of novel vinyl ester formulations derived from cardanol. Eur. Polym. J..

[8] Unnikrishnan K., Thachil E.T. (2008). Synthesis and characterization of cardanol-based epoxy systems. Des. Monomers Polym..

[9] Ionescu M., Petrović Z.S. (2011). Phenolation of vegetable oils. J. Serb. Chem. Soc..

[10] Jaillet F., Darroman E., Ratsimihety A., Auvergne R., Boutevin B., Caillol S. (2014). New biobased epoxy materials from cardanol. Eur. J. Lipid Sci. Technol..

[11] Jaillet F., Nouailhas H., Auvergne R., Ratsimihety A., Boutevin B., Caillol S. (2014). Synthesis and characterization of novel vinylester prepolymers from cardanol. Eur. J. Lipid Sci. Technol..

[12] Fouquet T., Puchot L., Verge P., Bomfim J.A., Ruch D. (2014). Exploration of cardanol-based phenolated and epoxidized resins by size exclusion chromatography and MALDI mass spectrometry. Anal. Chim. Acta.

[13] Campanella A., Fontanini C., Baltanas M.A. (2008). High yield epoxidation of fatty acid methyl esters with performic acid generated in situ. Chem. Eng. J..

[14] Kim Y.H., An E.S., Park S.Y., Song B.K. (2007). Enzymatic epoxidation and polymerization of cardanol obtained from a renewable resource and curing of epoxide-containing polycardanol. J. Mol. Catal. B Enzym..

[15] Chen J., Nie X., Liu Z., Mi Z., Zhou Y. (2015). Synthesis and application of polyepoxide cardanol glycidyl ether as biobased polyepoxide reactive diluent for epoxy resin. ACS Sustain. Chem. Eng..

[16] Radojčić D., Hong J., Ionescu M., Wan X., Javni I., Petrović Z.S. (2016). Study on the reaction of amines with internal epoxides. Eur. J. Lipid Sci. Technol..

[17] Park S.-J., Jin F.-L., Lee J.-R. (2004). Thermal and mechanical properties of tetrafunctional epoxy resin toughened with epoxidized soybean oil. Mater. Sci. Eng..

[18] Miao S., Wang P., Su Z., Zhang S. (2014). Vegetable-oil-based polymers as future polymeric biomaterials. Acta Biomater..

[19] Palmese G.R., McCullough R.L. (1992). Effect of epoxy–amine stoichiometry on cured resin material properties. J. Appl. Polym. Sci..

[20] Vanlandingham M., Eduljee R., Gillespie J. (1999). Relationships between stoichiometry, microstructure, and properties for amine-cured epoxies. J. Appl. Polym. Sci..

[21] Llevot A., Grau E., Carlotti S., Grelier S., Cramail H. (2016). From Lignin-derived Aromatic Compounds to Novel Biobased Polymers. Macromol. Rapid Commun..

[22] Wong O.A., Shi Y. (2008). Organocatalytic oxidation. Asymmetric epoxidation of olefins catalyzed by chiral ketones and iminium salts. Chem. Rev..

[23] Frohn M., Shi Y. (2000). Chiral ketone-catalyzed asymmetric epoxidation of olefins. Synthesis.

[24] Ager D.J., Anderson K., Oblinger E., Shi Y., VanderRoest J. (2007). An epoxidation approach to a chiral lactone: Application of the Shi epoxidation. Org. Process Res. Dev..

[25] McAninch I.M., Palmese G.R., Lenhart J.L., La Scala J.J. (2015). DMA testing of epoxy resins: The importance of dimensions. Polym. Eng. Sci..

[26] Hill L.W. (1997). Calculation of crosslink density in short chain networks. Prog. Org. Coat..

[27] Van Krevelen D.W., Te Nijenhuis K. (2009). Properties of Polymers: Their Correlation with Chemical Structure; Their Numerical Estimation and Prediction from Additive Group Contributions.

