# Supplementary material for: Influence of Epoxidized Cardanol Functionality and Reactivity on Network Formation and Properties
Source: Polymers (Basel). 2020 Aug 29;12(9):1956. doi: 10.3390/polym12091956 (PMC7563135; doi:10.3390/polym12091956)
Supplement: Supplementary file 1 [file polymers-12-01956-s001.pdf]

Influence of Epoxidized Cardanol Functionality and Reactivity on Network Formation and Properties

Emre Kinaci[1], Erde Can[2], John J. La Scala[3] and Giuseppe R. Palmese [1,*]

[1] Department of Chemical and Biological Engineering, Drexel University, Philadelphia, PA, 19104 USA; ek458@drexel.edu (E.K.); grp27@drexel.edu (G.R.P)

[2] Department of Chemical Engineering, Faculty of Engineering, Yeditepe University, Istanbul, Turkey; erde.can@yeditepe.edu.tr

[3] Army Research Laboratory, 4600 Deer Creek Loop, Aberdeen Proving Grounds, MD, 21005-5096, USA; john.j.lascala.civ@mail.mil

*** Correspondence: grp27@drexel.edu; Tel.: +1-215-895-5814 (G.R.P.)**

Supplementary Materials

The chemical shift assignments for the ^{1}H-NMR spectra shown in **Figure 1** are given in **Table S1.**

Table S1. ^{1}H-NMR Chemical shift assignments for CGE and SCEGE.

Annotated Peaks	δ (ppm)	Multiplicity	Number H
**CGE Spectrum (Figure 1a)**			
meta phenyl hydrogens (A)	7.18-7.22	t	2
orhto phenyl hydrogens (A)	6.71-6.83	m	2
Terminal double bond -CH= (B)	5.78-5.88	m	1
Internal double bond HC=CH (C)	5.31-5.47	m	2
Terminal double bond =CH_2 (D)	4.96-5.07	q	2
CH_2 between the double bonds (E)	2.71-2.76	q	2
CH_2 on the side chain next to phenyl ring (F)	2.54-2.61	t	2
CH_2 next to double bond (G)	1-54-1.63	s	2
CH_2 on the side chain (H)	1.57-1.64	t	2
Aliphatic -CH_2- (I)	1.31	s	2
Terminal -CH_3 (J)	0.86-0.95	m	3
CH_2-O-Ar (K)	3.91-4.22	q	2
CH-Oxirane (L)	3.33-3.38	m	1
CH_2-Oxirane(M)	2.89-2.92	t	2
**SCECGE Spectrum (Figure 1b) (only peaks with different assignments compared to CGE listed)**			
-CH_2 protons of the oxirane ring (c-c')	2.8-3.4	t	2
-CH_2 between the aliphatic oxirane (e)	1.71-1.79	m	2
-CH_2 next to aliphatic oxirane (g)	1.47-1.55	d	2

To estimate the cross-link density of the SCECGE-based networks and confirm the validity of our measurements, the simple method proposed by Hill [26] is used at full and incomplete epoxy-amine conversion. To simplify the calculations, the following is assumed: SCECGE is a tri-epoxy and an overall 75% epoxy conversion—corresponding to 60% secondary epoxy conversion—is calculated for the SCECGE epoxy system, and the density of the system is an assumed 1.12 g/cm^3. It is also assumed that SCECGE is a

tri-epoxy that resulted in a simple epoxy-amine system described as E3-A4 (E: epoxy, A: amine) for PACM and NX2003, and E3-A5 for DETA.

Table S2 shows the cross-link density (*v*) values of the SCECGE epoxy with different amines. The first column shows the *v* values calculated by assuming a full epoxy-amine network formation via Hill's method. The second column shows the *v* values corresponding to the 75% overall epoxy conversion as calculated via Hill's method as well. The final column also shows the *v* values as determined via DMA studies.

Table S2. Cross-link density values of SCECGE epoxy cured with different amines calculated via Hill's method and DMA studies.

**Amine adduct (w/ SCECGE)**	***v* (theoretical 100% conversion) (mol/m³)**	***v* (75% overall conversion) (mol/m³)**	***v* (obtained via DMA measurements) (mol/m³)**
PACM	1600	650	375
DETA	2000	800	500
NX2003	1300	520	355

The calculated *v* values for the ideally formed epoxy-amine network are almost three times higher than the network with 75% conversion, as shown in **Table S2**, suggesting that conversion of the epoxy and amines has a significant influence on the cross-link density of the formed network. In addition, the values calculated via DMA studies and via Hill's method for 75% epoxy conversion showed a better agreement, suggesting that the determined cross-link density values through DMA are valid and the partial network formation results in a much-lowered cross-link density than theoretical 100% conversion. In addition, the higher *v* values observed for the calculation method are likely due to assuming that SCECGE is tri-functional epoxy instead of having a real functionality of 2.45. Additionally, the rubbery elasticity relation is truly only valid for elastomers, and the assumptions used for that analysis do not necessarily hold for highly cross-linked thermosets, although past analysis has shown fairly good agreement. Second, the assumptions in the analysis by Hill assume all the functional groups on a monomer are equally reactive. That is not the case for the SCECGE system, where the primary glycidyl epoxies react to 99% while the secondary epoxides react to ~60%. These results show that the Hill analysis will overestimate the cross-link density for cured SCECGE-amine resins.

To check the validity of this method, the cross-link density values were also calculated for the DGEBA-amine systems via Hill's method, assuming an ideal network formation, and then compared with the cross-link density results obtained via DMA studies, which is presented in **Table S3** (E2-A4 and E2-A5 for PACM+NX2003 and DETA, respectively).

Table S3. Cross-link density values of DGEBA epoxy cured with different amines calculated via Hill's method and DMA studies.

**Amine adduct (w/ DGEBA)**	***v* (theoretical 100% conversion) (mol/m³)**	***v* (obtained via DMA measurements) (mol/m³)**
PACM	2300	2550
DETA	2600	3600
NX2003	1900	1800

The cross-link density values obtained through theoretical calculations and via experimental methods show good agreement for the DGEBA-amine systems, suggesting the validity of the Hill's equation for a diepoxy/diamine system. Differences between the experimental and calculated for the DGEBA-DETA system is probably due to the invalidity of the density assumption for this system and the error associated with rubbery elasticity for highly cross-linked thermosets.
